# After Experimental *Trypanosoma cruzi* Infection, Dying Hepatic CD3^+^TCRαβ^+^B220^+^ T Lymphocytes Are Rescued from Death by Peripheral T Cells and Become Activated

**DOI:** 10.3390/pathogens9090717

**Published:** 2020-08-31

**Authors:** Natalia Vacani-Martins, Marcelo Meuser-Batista, Otacilio C. Moreira, Cynthia Machado Cascabulho, Daniela Gois Beghini, Samuel Iwao Horita, Marcos Meuser Batista, Fernando Cleber Freitas, Juliana Rodrigues Guimarães, Andrea Henriques-Pons

**Affiliations:** 1 Laboratório de Inovações em Terapias, Ensino e Bioprodutos, Instituto Oswaldo Cruz, Fundação Oswaldo Cruz, Rio de Janeiro 21040-360, Brazil; natalia.vacani@bol.com.br (N.V.-M.); marcelomeuser@gmail.com (M.M.-B.); cynthiac@ioc.fiocruz.br (C.M.C.); beghini@ioc.fiocruz.br (D.G.B.); samuel.horita@ioc.fiocruz.br (S.I.H.); fcsfreitas.bio@gmail.com (F.C.F.); juliana.guimaraes@ioc.fiocruz.br (J.R.G.); 2 Depto de Anatomia Patológica e Citopatologia, Instituto Fernandes Figueira, Fundação Oswaldo Cruz, Rio de Janeiro 22250-020, Brazil; 3 Laboratório de Biologia Molecular e Doenças Endêmicas, Instituto Oswaldo Cruz, Fundação Oswaldo Cruz, Rio de Janeiro 21040-360, Brazil; otacilio@ioc.fiocruz.br; 4 Laboratório de Biologia Celular, Instituto Oswaldo Cruz, Fundação Oswaldo Cruz, Rio de Janeiro 21040-360, Brazil; meusermb@ioc.fiocruz.br

**Keywords:** hepatic T lymphocytes, liver immune response, CD3^+^B220^+^ T lymphocytes, *Trypanosoma cruzi* infection, cell death

## Abstract

The unusual phenotype of CD3^+^ T lymphocyte expressing B220, a marker originally attributed to B lymphocytes, was first observed in the liver of Fas/Fas-L-deficient mice as a marker of apoptotic T lymphocytes. However, other CD3^+^B220^+^ T lymphocyte populations were later described in the periphery as functional cytotoxic or regulatory cells, for example. Then, in this work, we studied whether hepatic CD3^+^B220^+^ T lymphocytes could play a role in experimental *Trypanosoma cruzi* infection. In control and infected mice, we observed two subpopulations that could be discerned based on CD117 expression, which were conventional apoptotic CD3^+^B220^+^(CD117^−^) and thymus-independent CD3^+^B220^+^CD117^+^ T lymphocytes. Regardless of CD117 expression, most B220^+^ T lymphocytes were 7AAD^+^, confirming this molecule as a marker of dying T cells. However, after infection, we found that around 15% of the CD3^+^B220^+^CD117^+^ hepatic population became B220 and 7AAD negative, turned into CD90.2^+^, and upregulated the expression of CD44, CD49d, and CD11a, a phenotype consistent with activated T lymphocytes. Moreover, we observed that the hepatic CD3^+^B220^+^CD117^+^ population was rescued from death by previously activated peripheral T lymphocytes. Our results extend the comprehension of the hepatic CD3^+^B220^+^ T lymphocyte subpopulations and illustrate the complex interactions that occur in the liver.

## 1. Introduction

Hepatic lymphoid and myeloid cells [1], such as Kupffer cells (KC) [2], dendritic cells, natural killer (NK) and natural killer T (NKT) cells, and γδ and αβ T lymphocytes, are anatomically distributed in the liver stroma and the lumen of sinusoids, a hepatic capillary system lined with liver sinusoidal endothelial cells (LSEC) [3]. Liver sinusoids are highly permeable fenestrated capillaries that allow the interaction of circulating leukocytes with sinusoidal cells and other cell types lying in the adjacent sinusoidal space (Disse space) [4]. All these subpopulations are exposed continuously to high concentrations of pathogen-associated molecular pattern (PAMP) molecules derived from the commensal microbiota [5] that reach the organ through the portal vein [1].

Naïve and antigen-primed peripheral T lymphocytes normally recirculate through the liver. However, apoptotic T lymphocytes are retained in the organ, become B220^+^ (CD45R) [6], a marker originally attributed to B lymphocyte, and die due to the Fas/Fas-L-dependent pathway. Apoptotic CD3^+^B220^+^ T lymphocytes were observed in high numbers in the liver, spleen, lymph nodes [7], and gut epithelium [8] of lpr/lpr (Fas-deficient) and gld/gld (Fas-L-deficient) mice. In these mice, the accumulation of CD3^+^B220^+^ T lymphocytes is associated with the development of lymphadenopathy and systemic autoimmunity. However, other authors observed in wild type mice and humans that apoptotic T lymphocytes express not only B220 but also subpopulations of functional effector T cells. For example, CD3^+^B220^+^CD4^−^CD8^−^ cells were described as highly cytotoxic lymphocytes against metastasized SL2 lymphoma cells after in vivo treatment with IL-2 and IL-12 [9]. The authors correlated the cytokine-based therapeutical success with the levels of this preexisting cytotoxic population that would be amplified after the treatment. Patients with T-cell large granular lymphocyte (T-LGL) leukemia and autoimmune lymphoproliferative syndrome (ALPS) showed the expansion of a CD3^+^ cytotoxic T lymphocyte population that expressed a B220 isoform [10]. Moreover, an extrathymic CD3^+^B220^low^CD90^low^CD4^−^CD8^−^ population was observed in the vaginal tract that utilizes less than half of the available T cell receptor (TCR) Vβ genes and is likely to play a role as a regulatory T cell [11].

Despite the importance of CD3^+^B220^+^ T lymphocytes in immunological responses and homeostasis, these cells are not well understood and are rarely studied regarding cellular ontogeny, survival signals, and effector functions, even in the case of diseases that affect liver function and lead to hepatic damage, as in Chagas disease after oral transmission. This parasitic disease, caused by *Trypanosoma cruzi* (*T. cruzi*) infection, is considered to have the most significant socioeconomic impact in Latin America, and an estimated six to seven million people are infected, mostly in Latin America [12]. The oral infection causes a more severe acute phase, with pronounced hepatomegaly, stromal cellular inflammatory infiltration, with infected KC and hepatocytes [13], and hepatic damage. Despite the number of cases due to oral infection having surpassed acute cases due to vector transmission, little is known about the liver cell populations that participate in both pathogenesis and infection control.

In this work, we observed in the murine livers of control mice two different subpopulations of CD3^+^B220^+^ T lymphocytes, which were discerned based on the expression or not of CD117 and biological characteristics. These subpopulations are cited here as the conventional apoptotic CD3^+^B220^+^ [6] T lymphocytes and a thymus-independent CD3^+^B220^+^CD117^+^ T lymphocyte population that we observed. Most B220^+^ T cells from both subpopulations were dead (7AAD^+^, annexin^+^, and TUNEL^+^ cells), confirming the expression of B220 as a marker of dying T lymphocytes in the liver [6].

After *T. cruzi* infection, there was an approximately ten-fold increase in the frequency of CD3^+^B220^+^ dying (7AAD^+^) conventional T lymphocytes in the liver. On the other hand, around fifteen percent of the CD3^+^B220^+^CD117^+^ T cells became 7AAD^−^ and B220^−^ after infection. Our results showed that the hepatic CD3^+^B220^+^CD117^+^ cells were rescued from death by infection-induced activated peripheral T lymphocytes. Moreover, these rescued T cells retained the expression of CD117, triggered the expression of CD90.2, and upregulated CD44, CD49d, and CD11a, assuming a phenotype of activated T cells. These results illustrate the complex intercellular interactions that take place in the liver and that much remains to be studied regarding the hepatic immune response.

## 2. Results

### 2.1. The Liver Is a Niche of CD3^+^CD4^−^CD8^−^B220^+^ T Lymphocytes

Aiming to optimize a method gentle enough to isolate cells that were triggering or undergoing apoptosis, hepatic T lymphocyte subpopulations were isolated by mechanical maceration, collagenase type II, or ready-to-use solutions of TrypLE or HyQTase (Figure S1). We observed no main differences regarding NK and NKT cells (Figure S1A–D) or γδ T lymphocytes (data not shown) using the different methods. However, there was an approximately five-fold enrichment in CD3^+^ T lymphocytes after mechanical maceration, varying from 2 × 10^7^ (Figure S1A) to 3.5–4 × 10^6^ cells when using the other methods (Figure S1B–D). Moreover, the CD3^+^ population isolated by maceration was enriched in CD4 CD8 double-negative T cells (Figure S1E–H), when comparing all methods. Based on these results, we decided to use the maceration method for the next experiments.

**Figure 1:**
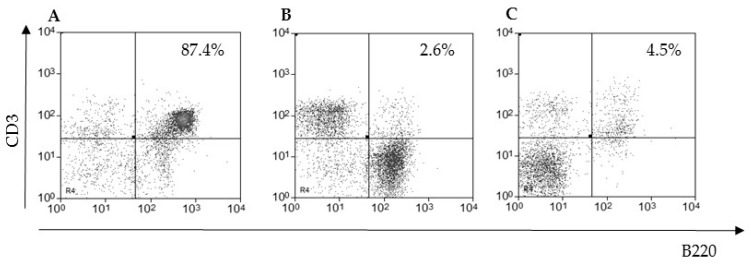
The liver as a site of CD3^+^B220^+^ T lymphocyte concentration. Thirteen-week old male C57BL/10 mice were perfused through the hepatic portal vein, and the cells were obtained by mechanical dissociation. The coexpression of CD3 and B220 was evaluated in leukocytes isolated from the liver (**A**), spleen (**B**), or thymus (**C**) and analyzed in the lymphocyte gate. The relative (percentage) number of cells per sample is indicated in each quadrant. Five mice were pooled per group in at least four independent experiments.

To evaluate whether the hepatic CD3^+^CD4^−^CD8^−^ T lymphocyte population expressed the B220 marker, we included this molecule in our panel and harvested lymphocytes from different anatomic sites to compare with the liver. We then observed that around 90% of the hepatic cells in the lymphocyte gate were CD3^+^B220^+^ (Figure 1A). This high concentration was only observed after liver maceration of at least thirteen-week-old male and female mice of all lineages tested, including CBA, AJ, BALB/c, and Swiss Webster (data not shown). On the other hand, only 2.6% of splenic (Figure 1B) and less than 5% of thymic (Figure 1C) T lymphocytes were CD3^+^B220^+^ in C57BL/10 mice.

We extended the phenotypic analysis of the hepatic CD3^+^B220^+^ population and observed that these cells were FoxP3^−^, CD90.2^−^, and CD62L^−^ (data not shown). 

### 2.2. Hepatic CD3^+^B220^+^ T Lymphocytes Are Thymus-Independent Cells that Express CD117 and TCR-αβ 

**Figure 2:**
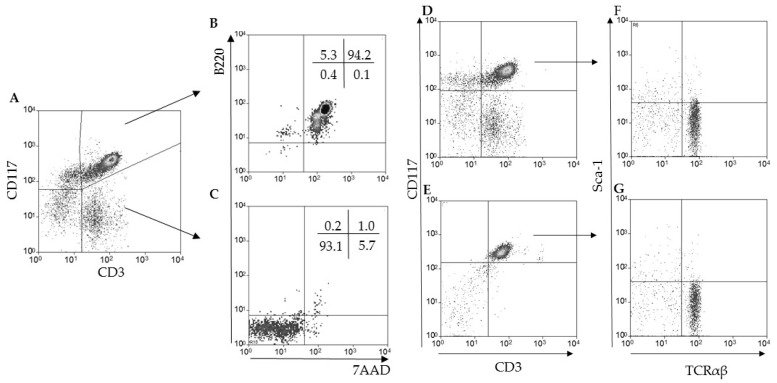
Hepatic CD3^+^B220^+^ T lymphocytes express CD117 and are thymus-independent. After liver perfusion through the hepatic portal vein, all cells were obtained from thirteen-week-old C57BL/10 (**A** to **C**), C57BL/6 (**D**,**F**), or nude/nude (**E**,**G**) mice by mechanical maceration. All samples were labeled using monoclonal antibodies for flow cytometry, and the analysis was done in the morphological gate of lymphocytes. The expression of B220 and 7AAD labeling was done as indicated by arrows (**B**,**C**), as TCRβ and Sca-1 (**F**,**G**). Five to seven mice were individually evaluated per group in at least ten (**A**–**C**) and two (**D**–**G**) independent experiments.

We then studied the ontogeny of the hepatic CD3^+^B220^+^ T lymphocyte population observed in older mice. As the murine liver has been described as having a hematopoietic function in adult mice, we included in our panel anti-CD117 (c-kit), the receptor for stem cell factor [14], and anti-stem-cell antigen-1 (Sca-1), a molecule associated with murine stem/progenitor cells. Moreover, we used athymic nude/nude mice [15] to evaluate whether these cells depended on the thymus for maturation and selective processes. When we combined the expression of all molecules during data analysis, we observed a major triple-positive population as CD3^+^B220^+^CD117^+^ (Figure 2A,B). More than 90% of the CD3^+^CD117^+^ T lymphocytes (Figure 2A) were dead B220^+^7AAD^+^ cells (Figure 2B) and around 5% were B220^+^7AAD^−^ (Figure 2B). Considering conventional CD3^+^CD117^−^ T lymphocytes (Figure 2A), we observed more than 90% of the events as B220 and 7AAD double-negative cells (Figure 2C). Only 1% of the conventional T cells were B220^+^7AAD^+^ (Figure 2C), a population phenotypically compatible with apoptotic T lymphocytes that die in the liver [6,7]. In regard to the evaluation of thymus dependence for the generation of CD3^+^B220^+^, we observed in wild type C57BL/6 counterparts that both subpopulations of CD3^+^ T lymphocytes (CD117^+^ and CD117^−^) were present in the liver (Figure 2D). On the other hand, as expected, conventional CD3^+^CD117^−^ T lymphocytes were not seen in nude/nude mice (Figure 2E), and more than 95% of the cells found in the lymphocyte gate were CD3^+^CD117^+^ cells (Figure 2E). Although nude/nude mice have some rare subpopulations of T lymphocytes, it is most likely that the ontogeny of CD3^+^B220^+^CD117^+^ T lymphocytes is thymus-independent. Moreover, we found no Sca-1 expression in CD3^+^B220^+^CD117^+^ cells in both mice groups, and all were TCR-αβ^+^ (Figure 2F,G).

### 2.3. Hepatic CD3^+^CD117^+^B220^+^ T Lymphocytes Are Rescued from Death and Downregulate B220 Expression after Experimental T. cruzi Infection

**Figure 3:**
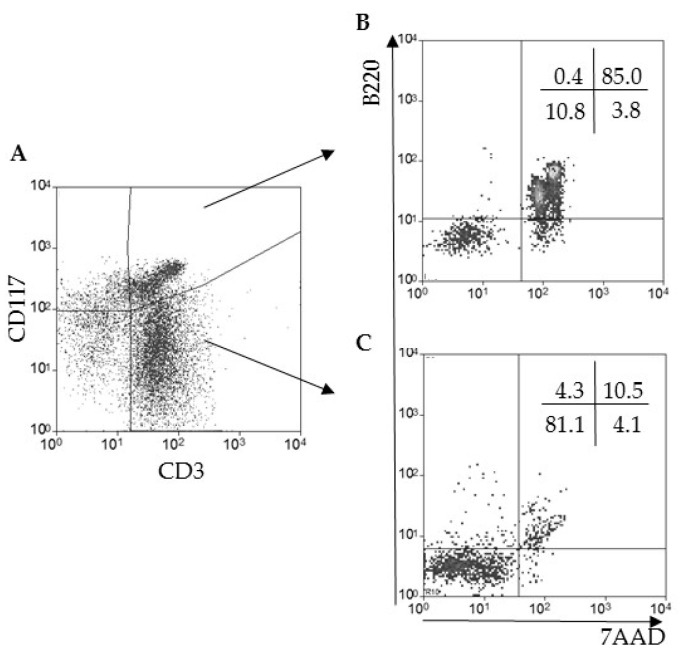
Part of the hepatic CD3^+^CD117^+^ T lymphocyte population downregulates the B220 marker after T.cruzi infection. The livers from thirteen-week-old C57BL/10 mice were perfused, and hepatic leukocytes were isolated by mechanical dissociation from *T. cruzi* infected mice (**A**). The analysis of CD3 and CD117 was done in the morphological gate of lymphocytes. The analysis of B220 expression and 7AAD labeling was done in CD3^+^CD117^+^ (**B**) or CD3^+^CD117^−^ (**C**) conventional T lymphocytes, as indicated by arrows. The percentage of positive events per quadrant is shown. Four to five mice per group were pooled in at least ten independent experiments.

Despite the expression of B220 and low viability, according to the 7AAD labeling (Figure 2B), we tested whether hepatic CD3^+^B220^+^CD117^+^ T lymphocytes could respond to antigen exposure and become activated, a primary biological function of T cells. We then infected C57BL/10 mice with *T. cruzi* and observed, in the gate of CD3^+^CD117^+^ T lymphocytes (Figure 3A), that most cells remained B220^+^7AAD^+^ (Figure 3B). However, around 10% of the events were B220^−^7AAD^−^ cells (Figure 3B), an almost 30-fold increase when compared with uninfected mice (Figure 2B). This result suggested that part of the hepatic CD3^+^CD117^+^ T lymphocyte population could be rescued from death while losing the expression of B220. Regarding conventional CD3^+^CD117^−^ T lymphocytes, there was an increase after the infection (Figure 3A), when compared with control mice (Figure 2A), a known T lymphocyte expansion induced by the infection [16]. Around 85% of this population was 7AAD^−^ (Figure 3C upper left and lower left quadrants). Moreover, around 10% of the cells acquired the B220 expression on the cell membrane and became 7AAD^+^ (Figure 3C), suggesting an increase in conventional apoptotic T lymphocyte death in the liver when compared with control mice (Figure 2C). 

### 2.4. Rescued CD3^+^CD117^+^ T Lymphocytes Upregulate CD44 (CD44^high^) and Downregulate PD1 Expression after T. cruzi Infection

**Figure 4:**
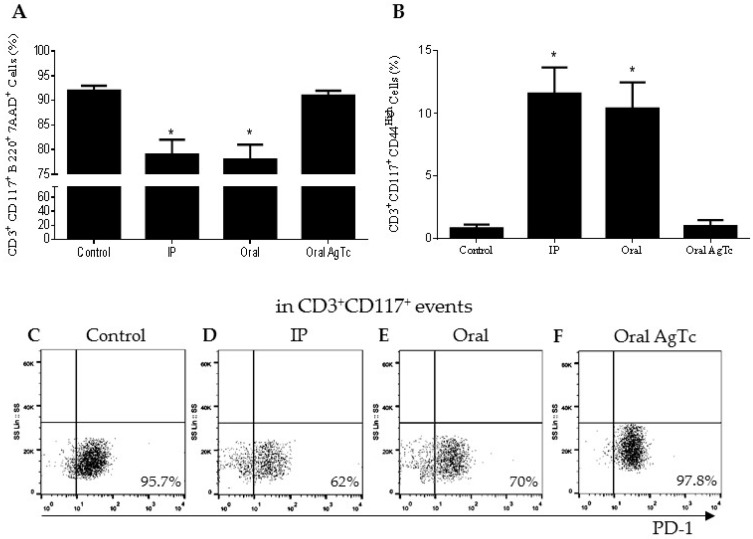
Rescued CD3^+^CD117^+^ T cells become CD44^high^ after *T. cruzi* infection. Thirteen-week-old C57BL/10 mice were grouped as control uninfected mice; mice infected with *T. cruzi* Y strain through the intraperitoneal (**IP** group) or oral (in the oral cavity) (**oral** group) routes, and mice treated with a single dose of *T. cruzi* extract by gavage (**oral AgTc** group). After fifteen days, hepatic leukocytes were obtained by mechanical maceration after liver perfusion and total CD3^+^B220^+^CD117^+^7AAD^+^ (**A**) and CD3^+^CD117^+^CD44^high^ (**B**) cells were evaluated. PD1 expression was also evaluated in CD3^+^CD117^+^ T lymphocyte gate (**C**–**F**) in the same groups. Five independent experiments were done, using at least five mice pooled per group. * represents *p* < 0.05 when infected groups were compared with control and oral AgTc groups. The bars represent mean values.

To better understand the survival stimuli for CD3^+^B220^+^CD117^+^ T lymphocytes and phenotypic shift to CD3^+^CD117^+^ after *T. cruzi* antigen exposure, we infected mice through different routes (oral and IP) and administered *T. cruzi* extract by gavage (oral AgTc) (Figure 4). The administration by gavage was done to simulate what happens when the hepatic stroma is exposed to antigens of intestinal origin, a known inducer of a more tolerant liver immune response [1]. In control mice, we observed once more that at least 90% of the events in the lymphocyte gate were CD3^+^B220^+^CD117^+^7AAD^+^ (Figure 4A). On the other hand, there was a reduction after IP or oral infection, and around 78% of the events were CD3^+^B220^+^CD117^+^7AAD^+^ (Figure 4A, IP and oral). Moreover, when parasite extract was administered by gavage, around 90% of the cells remained 7AAD^+^, similar to the control group (Figure 4A). Once T lymphocytes upregulate the expression of CD44 after antigen/MHC engagement and become effector or memory T cells [17], we evaluated the expression of this molecule in CD3^+^CD117^+^ liver T lymphocytes. We excluded the B220 labeling from the gating strategy because we observed that, after activation, these cells lose this marker. In control and oral AgTc groups, only 1–3% of the cells were CD44^high^ (Figure 4B), while in the IP and oral infected groups, 10–14% of the cells upregulated this molecule (Figure 4B). Around 98% of the CD3^+^CD117^+^ T lymphocytes that were CD44^high^ were also B220^−^ and 7AAD^−^ (data not shown). 

We evaluated another molecule associated with T lymphocyte activation, which is programmed cell death protein 1 (PD-1). Although this molecule is transiently expressed on the cell membrane after TCR stimulation, its constitutive expression plays a role in limiting the physiological immune responses [18], as in the case of the liver. We observed that more than 95% of the hepatic CD3^+^CD117^+^ T lymphocytes expressed PD-1 in the control and oral AgTc groups (Figure 4C,F). Although, after the infection, most CD3^+^CD117^+^ T lymphocytes remained PD-1^+^, there was a reduction to 62% and 70% in the IP and oral groups, respectively (Figure 4D,E). 

**Figure 5:**
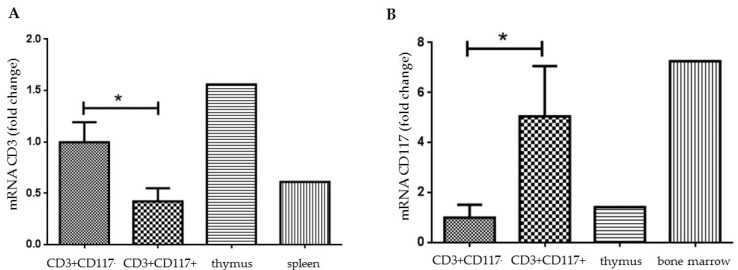
CD3 and CD117 gene expression in hepatic T lymphocytes. Thirteen-week-old C57BL/10 mice were inoculated with *T. cruzi* extract and activated conventional CD3^+^CD117^−^B220^−^7AAD^−^CD44^high^ or CD3^+^CD117^+^B220^−^7AAD^−^CD44^high^ hepatic T lymphocytes were purified from the liver by flow cytometry. CD3 (**A**) and CD117 (**B**) gene expression in purified T lymphocytes were evaluated by real-time quantitative PCR. Thymus, spleen, and bone marrow samples were used as controls for the PCR reaction. Each bar represents the mean +/− SD of five individual animals per group. * represents *p* < 0.05.

We confirmed by PCR the transcription of the CD117 and CD3 molecules in purified activated lymphocytes isolated from mice IP injected and boosted with *T. cruzi* extract. The livers were macerated and viable CD3^+^CD117^−^CD44^high^CD62-L^−^ (conventional) and CD3^+^CD117^+^CD44^high^CD62-L^−^ (CD3^+^CD117^+^) cells, both B220^−^ populations, were simultaneously sorted. We also used fragments of the spleen, thymus, and bone marrow as internal PCR controls (Figure 5A,B). We observed that conventional T lymphocytes isolated from the liver stroma express higher levels of CD3 mRNA when compared with CD3^+^CD117^+^ cells (Figure 5A). The flow cytometry data analysis did not parallel this result. On the other hand, we found a five- to seven-fold increase in CD117 gene transcription in CD3^+^CD117^+^ cells (Figure 5B), when compared with conventional T lymphocytes. 

### 2.5. Rescued CD3^+^CD117^+^ T Lymphocytes Upregulate the Expression of Molecules Involved in Cellular Migration, Adhesion, Costimuli, and Activation after T. cruzi Infection

**Figure 6:**
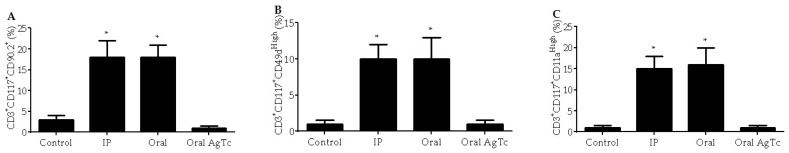
CD3^+^CD117^+^ T lymphocytes upregulate molecules involved in migration, adhesion, costimuli, and activation after *T. cruzi* infection. Thirteen-week-old C57BL/10 mice were infected with *T. cruzi* Y strain through the IP (IP group) or oral (in the oral cavity) (oral group) routes or parasite extract was administered by gavage (oral AgTc group). After fifteen days, hepatic leukocytes were obtained by mechanical dissociation, and the expression of CD90.2 (**A**), CD49d^high^ (**B**), and CD11a^high^ (**C**) was evaluated in the CD3^+^CD117^+^ T lymphocyte gate. Five independent experiments were done using five mice pooled per group. * represents *p* < 0.05 when infected mice were compared with control and oral AgTc groups. The bars represent mean values.

We further explored the phenotypic shift of CD3^+^B220^+^CD117^+^ T lymphocytes when they become activated and are rescued from death and used the same groups of mice and the gating strategy that excluded the B220 marker. We found only 2 to 4% of CD3^+^CD117^+^CD90.2^+^ in the control group and mice orally administered with parasite extract (oral AgTc group) (Figure 6A). On the other hand, after infection (IP or oral), there were around 18% of CD3^+^CD117^+^CD90.2^+^ cells (Figure 6A). We included CD90.2 as a pan T lymphocyte marker, although it has also been described in neurons, fibroblasts, several cancer cells [19], and even as a marker of hematopoiesis [20]. To our surprise, and even though CD3^+^CD117^+^ T lymphocytes express TCRαβ, these cells expressed CD90.2 only after infection. Moreover, CD3^+^CD117^+^ T lymphocytes were CD49d^low^ and CD11a^low^ in control mice (data not shown), but after infection, both molecules were upregulated, and we found around 10% of the cells as CD3^+^CD117^+^CD49d^high^ (Figure 6B) and around 15% as CD3^+^CD117^+^CD11a^high^ (Figure 6C) T lymphocytes. 

### 2.6. CD3^+^B220^+^CD117^+^ T Cells Are Not Rescued from Death after Acetaminophen-Induced Hepatic Damage 

We then studied whether liver CD3^+^CD117^+^ T lymphocytes would engage in damage-associated molecular pattern (DAMP) recognition and also undergo activation. We first induced hepatocyte damage by treating mice with acetaminophen and used a dose sufficient to cause cell necrosis (Figure S2A), with 100% of mice survival rate (Figure S2B). However, fifteen days after the treatment, most liver CD3^+^CD117^+^ T lymphocytes remained B220^+^7AAD^+^ and were not rescued from death (Figure S2C,D).

### 2.7. Liver CD3^+^CD117^+^B220^+^ T Cells Are Rescued from Death and Acquire Effector/Effector Memory Phenotype (CD44^high^) through a Peripheral T Lymphocyte-Dependent Way

**Figure 7:**
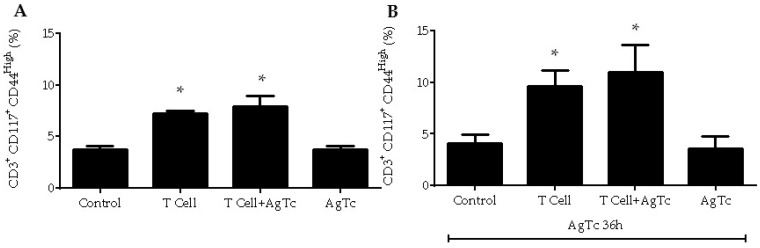
Peripheral T lymphocyte-dependent rescue of hepatic CD3^+^B220^+^CD117^+^ T cells and activation. Activated splenic T lymphocytes (CD3^+^CD117^−^CD44^high^CD197^−^) were purified by flow cytometry from mice previously injected with *T. cruzi* extract plus adjuvant through the IP route. Then, 5 × 10^4^ purified cells were transferred to each recipient mouse according to the following groups: “control”, untreated mice; “T cell”, mice that received only activated splenic T lymphocyte; “T cell+AgTc”, mice that received activated splenic T lymphocytes and (simultaneously) *T. cruzi* extract by gavage, and “AgTc”, mice that received only *T. cruzi* extract by gavage. The mice received extract equivalent to 1 × 10^7^ parasites per mouse by gavage. Fifteen days after the treatments, all groups were either euthanized for liver leukocyte analysis (**A**) or *T. cruzi* extract by gavage for liver cells analysis after 36 h (**B**). Five independent experiments were done using five mice pooled per group. * represents *p* < 0.05 when the groups T cell or T cell+AgTc were compared with control and AgTc groups. The bars represent mean values.

Taken together, our results indicated that at least part of the liver CD3^+^B220^+^CD117^+^ T lymphocyte population could be rescued from death after infection, downregulating the expression of B220, remaining CD117^+^, and assuming a phenotype of viable activated T cells. We published recently that, in the acute phase of experimental *T. cruzi* infection, activated peripheral T lymphocytes induce the activation of stromal hepatic T lymphocyte and lead to a local proinflammatory immune response [21]. Thus, we studied whether activated peripheral T lymphocytes could rescue hepatic CD3^+^B220^+^CD117^+^ T lymphocytes from death and induce cellular activation. We then IP injected and boosted donor mice with *T. cruzi* antigens (parasite extract) and purified splenic CD3^+^CD117^−^CD44^high^CD197^−^ (conventional effector/effector memory) T lymphocytes. The experimental groups then consisted of recipient mice that IP received only activated splenic T lymphocytes (T cell group); mice that received activated splenic T lymphocytes and parasite extract by gavage (T cell + AgTc); mice that received only parasite extract by gavage (AgTc), and control untreated individuals. All mice were euthanized after fifteen days, and we found around 4% of CD3^+^CD117^+^CD44^high^ activated T lymphocytes in the livers of control and AgTc groups of mice (Figure 7A). However, when conventional activated splenic T lymphocytes were adoptively transferred, we found 7 to 9% of CD3^+^CD117^+^CD44^high^ (B220^−^7AAD^−^) T lymphocytes in both T cell and T cell+AgTc groups (Figure 7A). 

As an antigenic challenge could increase the levels of CD3^+^B220^+^CD117^+^ T lymphocyte rescue and activation, we repeated the experiment and, fifteen days after the cell transfer and/or antigen administration, all mice received *T. cruzi* extract by gavage. After thirty-six hours (Figure 7B), we observed a discrete increase in CD3^+^CD117^+^CD44^high^ cells (B220^−^7AAD^−^) in both groups that received activated splenic T lymphocytes (Figure 7B), varying from 10 to 13% of cells. Once more, only around 4% of CD3^+^CD117^+^CD44^high^ cells were found in the other groups (Figure 7B). It is unlikely that the transferred activated splenic T lymphocytes were collected in the liver and responsible for the increase in CD3^+^CD117^+^CD44^high^ T lymphocytes in the organ, as sorted cells were CD117^−^ and only 5 × 10^4^ activated cells were transferred per mouse.

### 2.8. Rescued CD3^+^CD117^+^ T Lymphocytes Are Derived from Liver-Resident CD3^+^B220^+^CD117^+^ Cells

**Figure 8:**
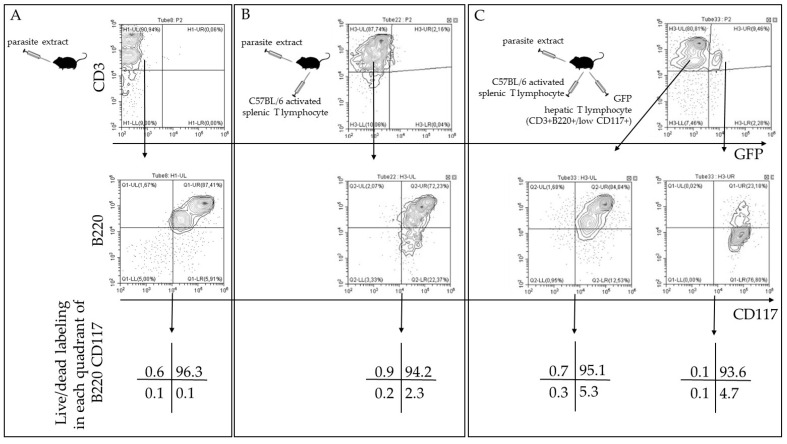
Activated CD3^+^CD117^+^B220^−^ cells are derived from CD3^+^B220^+/low^CD117^+^ T lymphocytes in the liver. Activated splenic T lymphocytes (CD3^+^CD117^−^CD44^high^CD197^−^) were purified from wild type C57BL/6 mice IP injected and boosted with *T. cruzi* extract. At the same time, liver CD3^+^B220^+/low^CD117^+^ T cells were purified from untreated syngeneic C57BL/6 Tg14 (act-EGFP) OsbY01 mice and experimental groups were as follows: mice that received only parasite extract by gavage (**A**); mice that adoptively received activated splenic T lymphocytes (5 × 10^5^ cells/mouse) and parasite extract (**B**), and mice that received activated splenic T lymphocytes plus hepatic CD3^+^B220^low/+^CD117^+^ T lymphocytes purified from unstimulated C57BL/6 Tg14 (act-EGFP) OsbY01 mice (1 × 10^4^ cells/mouse) and parasite extract (**C**). After two weeks, recipients’ liver lymphocytes were analyzed by flow cytometry, and CD3^+^ cells were evaluated in the morphological gate of lymphocytes. The analysis of B220 and CD117 labeling was done in the lymphocyte gate and the gate of CD3^+^ cells. Finally, the analysis of dead cells (positive events for live/dead labeling) was done for each quadrant of the B220 and CD117 labeling. Two experiments were done, with six mice individually evaluated per group.

At this point, we wanted to confirm whether activated hepatic CD3^+^CD117^+^CD44^high^ (B220^−^7AAD^−^) T lymphocytes were indeed rescued cells generated from the CD3^+^B220^+^CD117^+^ population or some other subpopulation. To this aim, we used C57BL/6 Tg14 (act-EGFP) OsbY01 mice as donors of hepatic CD3^+^B220^+^CD117^+^ cells expressing the green fluorescent protein (GFP). The groups were then separated as follows: control mice (Figure 8A); mice that adoptively received activated splenic T lymphocytes (5 × 10^5^ cells/mouse) (Figure 8B), and mice that received activated splenic T lymphocytes plus hepatic CD3^+^B220^low/+^CD117^+^ T lymphocytes purified from unstimulated C57BL/6 Tg14 (act-EGFP) OsbY01 mice (1 × 10^4^ cells/mouse) (Figure 8C). All groups received parasite extract by gavage.

When only *T. cruzi* extract was given by gavage, we observed in the CD3^+^ T lymphocyte gate that around 87% of the events were B220^+^CD117^+^ and 96% of these cells were dead (Figure 8A), as previously seen. On the other hand, after the adoptive transfer of activated splenic T lymphocytes, the analysis in the gate of CD3^+^ T lymphocytes showed two distinct populations, which were around 72% of B220^+^CD117^+^ and 22% of B220^−^CD117^+^ T cells (Figure 8B). In the former population, more than 94% of the cells were dead, and in the B220^−^ population, only around 2% of the cells were live/dead^+^ (Figure 2B), also as we previously observed. When we adoptively transferred activated splenic T lymphocytes from wild type mice and hepatic CD3^+^B220^low/+^CD117^+^ T lymphocytes from C57BL/6 Tg14 (act-EGFP) OsbY01 mice (Figure 8C), we observed around 80% of CD3^+^GFP^−^ cells in the lymphocyte morphological gate of the recipient mice (Figure 8C). In this gate, we found once more two populations, which were 75% of B220^+^CD117^+^ (mostly dead) and around 23% of B220^−^CD117^+^ (mostly live/dead^−^ cells) (Figure 8C). Considering the transferred hepatic T lymphocytes (GFP^+^), the main population, we observed around 9.5% of CD3^+^GFP^+^ T cells in the lymphocyte gate. Around 23% of the GFP^+^ cells were B220^+^CD117^+^ (mostly live/dead^+^ cells) (Figure 8C), and there was an enrichment of GFP^+^ cells as B220^−^CD117^+^ T cells, with less than 5% of dead cells. This result confirms that rescued CD3^+^B220^−^CD117^+^ viable cells are derived from CD3^+^B220^low/+^CD117^+^ hepatic T lymphocytes. When only hepatic CD3^+^B220^low/+^CD117^+^ T lymphocytes were collected from C57BL/6 Tg14 (act-EGFP) OsbY01 mice and transferred to recipient mice, without activated splenic T cells, more than 90% of the GFP^+^ cells remained B220^+^ live/dead^+^ cells (data not shown). Our data indicate that, despite the expression of B220, a considered apoptosis T cell marker in the liver, a subpopulation of thymus-independent hepatic CD3^+^B220^+^CD117^+^ T lymphocytes was rescued from death and became activated, assuming the phenotype CD3^+^B220^−^CD117^+^CD90.2^+^CD11a^high^CD49d^high^CD44^high^. 

## 3. Discussion

This work started with a long-lasting series of experiments aiming to optimize the isolation of T lymphocyte subpopulations expressing B220 from the hepatic stroma. The maceration method was gentle enough to isolate cells that were not only 7AAD^+^ but also Annexin V and TUNEL positive cells (data not shown). These cells are very delicate and are easily lost during enzymatic digestion procedures. Due to a sequence of experimental precautions, we enriched the samples in CD3^+^B220^+^ and 7AAD^+^ cells that are not usually observed in wild type mice.

**Figure 9:**
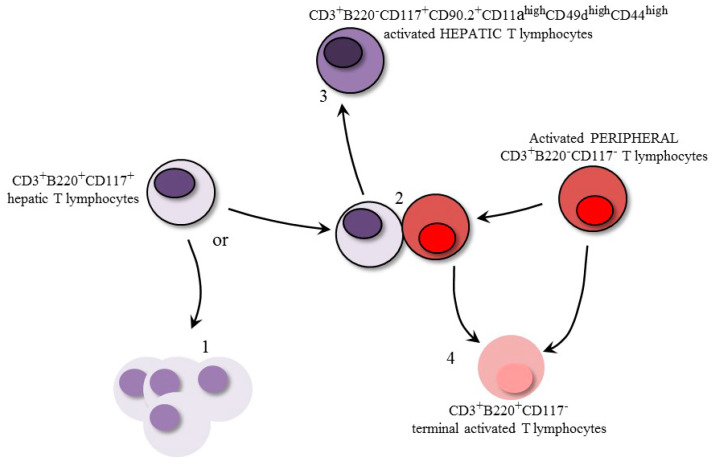
Illustrated summary of the CD3^+^B220^+^ T lymphocyte subpopulations found in the liver and cellular interactions. The hepatic CD3^+^B220^+^CD117^+^ population proceeds to inexorable death (**1**) unless rescued by a previously activated peripheral T lymphocyte (**2**). It is still unknown if this is a lymphocyte:lymphocyte direct interaction (as represented) or if it is mediated by an antigen-presenting cell. Then, the rescued CD117^+^ T lymphocytes will assume a phenotype compatible with activated T cells as CD3^+^B220^−^CD117^+^CD90.2^+^CD11a^high^CD49d^high^CD44^high^, losing the expression of B220 (**3**). The peripheral activated T lymphocytes will eventually become B220^+^ and die (**4**), as previously described, either after interacting with CD3^+^B220^+^CD117^+^ T cells or not.

Based on our present results, we believe that the CD3^+^B220^+^ T lymphocyte populations found in the murine liver are composed of conventional peripheral T lymphocytes that become B220^+^ in the liver before death (CD117^−^), as published before, and a thymus-independent T lymphocyte (CD117^+^) population (Figure 9). In regard to the CD3^+^B220^+^CD117^+^ cells, we believe that they will encounter one of two possible fates, which are inexorable death or cellular rescue by a previously activated peripheral T lymphocyte (Figure 9). In any case, these hepatic cells retain CD117 expression, like other mature cells of the immune system—as mast cells [22], for example.

It is interesting how dying CD3^+^B220^+^CD117^+^ T lymphocytes, with exposed phosphatidylserine (PS) as TUNEL^+^ events (data not shown), accumulate in the hepatic stroma, not being rapidly phagocytosed. This is different from the thymus, where dying thymocytes are promptly removed from the tissue. We do not know through which mechanisms phagocytic cells in the liver “ignore” the dying CD3^+^B220^+^CD117^+^ T lymphocytes. On the other hand, we can speculate that the exposure of PS on these cells in the liver may contribute to the intrinsic immunological tolerance observed in the organ. It is known that the engagement of PS to PS receptor over phagocytic cells leads to the secretion of IL-10, TGF-β, and leukotrienes that downregulate effector functions in the immune system [23]. Much remains to be elucidated about CD3^+^B220^+^CD117^+^ T lymphocytes and mainly about activated CD3^+^B220^−^CD117^+^ T cells in the liver.

Although most of the CD3^+^B220^+^CD117^+^ T lymphocytes were dying 7AAD^+^ cells, we investigated whether part of this population could be rescued from death. After *T. cruzi* infection or the adoptive transfer of activated splenic T lymphocytes, we observed around 15% of the hepatic CD3^+^B220^+^CD117^+^ T lymphocyte population becoming B220^−^7AAD^−^ and CD44^high^CD90.2^+^CD49d^high^CD11a^high^. These molecules mediate T lymphocyte migration, adhesion, survival, activation, intercellular interactions, and other primordial functions that mediate full lymphoid activity in a mature immune system [24,25,26]. The rescued activated CD3^+^CD117^+^ T lymphocytes are molecularly equipped, and we believe that they are capable of exerting effector functions either in the liver itself or in the periphery.

We started this work aiming to study hepatic subpopulations of CD3^+^B220^+^ T lymphocytes that could play a role in *T. cruzi* infection. However, to our surprise, the hepatic CD3^+^B220^+^(CD117^+^) population that we found loses the B220 expression after death rescue and activation. Therefore, this subpopulation is not likely to compose the peripheral B220^+^ T lymphocyte populations described before as important cells in other pathologies [9,10]. Still, activated CD3^+^CD117^+^ T cells, derived from the CD3^+^B220^+^CD117^+^ population, may exert important effector functions, and we are investigating whether these rescued cells are proinflammatory. We believe that this is possible because some of these cells downregulate PD-1 expression after activation, a known regulatory molecule that depresses T lymphocyte function. In regard to *T. cruzi* infection, these cells may contribute to restraining liver parasitism, for example, like other non-classic lymphoid cells described before [16,27,28].

## 4. Materials and Methods

### 4.1. Mice

Thirteen-week-old specific pathogen-free (spf) male C57BL/10 mice were obtained from the FIOCRUZ animal facility (ICTB). Nude/nude (C57BL/6 background) and syngeneic counterparts were purchased from Biotério Central of the Universidade de São Paulo. Dr. Regina Coeli Goldenberg and Dr. Isalira Ramos, from the Instituto de Biofísica Carlos Chagas Filho at the Universidade Federal do Rio de Janeiro, kindly provided C57BL/6 Tg14 (act-EGFP) OsbY01 mice. Except for nude/nude mice, all mouse lineages were housed for at least one week before experimentation at the Divisão de Experimentação Animal at the Laboratório de Inovações em Terapias, Ensino e Bioprodutos—Instituto Oswaldo Cruz, Fiocruz. All procedures were performed following the “Guide for the Care and Use of Laboratory Animals” (DHEW Publication No. (NIH) 80-23, revised 1985). The FIOCRUZ Committee of Ethics in Research approved this project (L006/15), according to resolution 196/96 of the National Health Council of the Brazilian Ministry of Health.

### 4.2. Liver Cells Isolation

The livers were perfused through the portal vein using Dulbecco’s modified eagle medium (DMEM) (Life Technologies, Bedford, MA, USA), in the absence of fetal bovine serum (FBS), to remove blood contaminants. The hepatic lymphoid cells were then obtained through the perfusion of the liver with 5 mL of HyQtase (GE Healthcare, Freiburg, Germany) or TrypLE Express (Thermo Fisher) at 37 °C, as indicated in the figure legends. After four to five cleavages per hepatic lobe, the organs were subjected to the previous dissociation solution at 37 °C in a Petri dish under gentle agitation for 10 min. For collagenase dissociation, the livers were perfused, fragmented, and incubated under gentle agitation for 15 min at 37 °C (120 U/mL type II collagenase) (Worthington, NJ, USA). The enzyme was previously diluted in DMEM containing HEPES 10mM (Thermo Fisher) and CaCl_2_ 3mM (Sigma Aldrich, St. Louis, Mo, USA). Unless otherwise indicated, all results were obtained by maceration. 

Regardless of the isolation method, all samples were filtered using a 40 μm strainer (Greiner Bio-One, Frickenhausen, Germany) and washed in DMEM containing 10% of FBS (Thermo Fischer). The samples were then centrifuged at 237× *g* for 10 min at 4 °C, the supernatants were discarded, and the pellet was resuspended in 1 mL of DMEM containing 10% of FBS. For flow cytometry, 10% of inactivated sheep serum was used to block Fcγ receptors, and all cells were maintained in ice until use.

### 4.3. Splenic Lymphocyte Isolation

The spleens were collected, macerated, and the cells were centrifuged twice at 237× *g* for 10 min at 4 °C in DMEM containing 10% of FBS. Pelleted cells were resuspended in 1 mL of red blood cells lysis solution (10% PBS (Thermo Fischer) in distilled water) for ten seconds, and 10 mL of 1× PBS was added. All cells were washed, resuspended in 1 mL of the FcγR blocking solution, and maintained in ice until antibody labeling.

### 4.4. Acetaminophen Treatment

C57BL/10 mice were intraperitoneally (IP) injected with PBS or with a single dose of acetaminophen (500, 750, or 1000 mg/Kg) (Medley, SP, Brazil). Twelve hours after the injection, blood samples were collected in heparinized tubes, centrifuged (237× *g* for 8 min), and transaminase (ALT) activity was evaluated according to the manufacturer’s orientations (Labtest, MG, BR). Individual mortality was determined daily.

### 4.5. T. cruzi Infection

Bloodstream trypomastigote forms of *T. cruzi* Y strain were obtained from Swiss-Webster mice after seven days of infection. The parasites were counted, and the inoculum for experimental infection was adjusted in PBS. As mentioned in the figure legends, the animals were divided into the following groups: “control”, which received one IP injection of PBS; “IP”, IP infection using 1 × 10^4^ trypomastigote forms of *T. cruzi*; “oral”, oral infection using 1 × 10^7^ parasites in the oral cavity; “oral AgTc”, antigen (parasite extract) of *T. cruzi* administered by gavage at a dose equivalent to 1 × 10^7^ parasites per mouse. The parasite extract was prepared by three heating cycles at 60 °C for 10 min, followed by one cycle of freezing and thawing. 

### 4.6. Flow Cytometry

Isolated cells were counted in a Neubauer chamber and distributed in 96-well round-bottom plates (3 × 10^5^ hepatic or 1 × 10^6^ splenic cells per well) for monoclonal antibody labeling. All antibodies were previously titrated and purchased from BioLegend (San Diego, CA, USA). Cell viability was assessed by 7AAD (BioLegend) labeling or using the Live/Dead cytometric kit (Thermo Fisher) according to the manufacturers’ instructions. The samples were acquired using a Cyan ADP or a Cytoflex S flow cytometer (Beckman Coulter, Brea, CA, USA) at the Multiparametric Multiuser Flow Cytometry Facility at Instituto Oswaldo Cruz. Data were analyzed using Summit (version 6.1), Cytexpert (version 1.1.10), or FlowJo (version 10.0) computer program. Doublets exclusion was done using FSC-H × FSC-A dot plots, and CD3-based retrogates were used to define the morphological lymphocyte region.

### 4.7. Adoptive Transfer of T Lymphocytes

#### 4.7.1. Single Transfer (Activated Splenic T Lymphocytes)

Donor C57BL/10 mice received weekly one IP injection of *T. cruzi* extract (equivalent to 1 × 10^7^ parasites per mouse) in 6 mg of aluminum hydroxide (AlOH) (Sigma-Aldrich) for two weeks. Seven days after the second injection, activated splenic T lymphocytes (CD3^+^CD117^−^CD44^high^CD197^−^) were purified by flow cytometry using a MoFlo Astrius (Beckman Coulter) flow cytometer at the Multiuser Cell Sorting Facilities at the Instituto Oswaldo Cruz. Only samples with at least 95% purity and viability were used. Experimental groups were then divided according to parasite extract oral administration (equivalent to 1 × 10^7^ parasites) and IP cellular transfection of purified splenic cells (5 × 10^4^ cells/mouse) to syngeneic mice, as shown in Table 1: 

After fifteen days, all mice were challenged with *T. cruzi* extract by gavage (equivalent to 1 × 10^7^ parasites) and euthanized after 36 h, as indicated in the figure legends.

#### 4.7.2. Double Cellular Transfer (Activated Splenic and Unstimulated Hepatic T Lymphocytes) 

Donor C57BL/6 mice received weekly one IP injection of *T. cruzi* extract in AlOH for two weeks and activated splenic T lymphocytes (CD3^+^CD117^−^CD44^high^CD197^−^) were purified by flow cytometry. Liver CD3^+^B220^+/low^CD117^+^ cells were purified from non-injected syngeneic C57BL/6 Tg14 (act-EGFP) OsbY01 mice by flow cytometry using a MoFlo Astrius or an Aria flow cytometer (IOC Multiuser Facilities of Flow Cytometry). The experimental groups of recipient mice are shown in Table 2. 

Individual recipient mice received parasite extract by gavage at a dose equivalent to 1 × 10^7^ parasites. For adoptive T lymphocyte transfer, 5 × 10^5^ activated splenic and/or 1 × 10^4^ hepatic T lymphocytes were injected. After fifteen days, hepatic lymphocytes were collected from all groups of recipient mice and analyzed by flow cytometry. 

### 4.8. Real-Time Quantitative PCR

Thirteen-week old C57BL/10 mice were injected with *T. cruzi* extract, as indicated elsewhere in this work, and hepatic activated CD3^+^CD117^−^B220^−^7AAD^−^CD44^high^ or CD3^+^CD117^+^B220^−^7AAD^−^CD44^high^ T lymphocytes were purified by flow cytometry. Thymus, spleen, and bone marrow samples were also obtained and used as controls for the PCR reaction. Total mRNA was extracted using the RNeasy kit (Qiagen) and treated with DNase to exclude DNA contamination. RNA was converted to cDNA by reverse transcription using the high-capacity cDNA kit, as recommended by the manufacturer (Applied Biosystems, Foster City, CA, USA). Real-time quantitative PCR was performed on a ViiA 7 Real-Time PCR System using TaqMan Fast Advanced Master Mix (Applied Biosystems) and Taqman Gene Expression Assay Primers. We used the Probes System to examine CD3e (Mm00599683_m1), CD117 (Mm00445212_m1), glyceraldehyde 3-phosphate dehydrogenase (GAPDH) (Mm99999915_g1), and b actin (Mm00504274_m1), following the manufacturer’s protocols. Each of these primer sets gave a unique product, and PCR assays were done in triplicate. Sample quantification was obtained by the relative standard curve normalized by GAPDH and confirmed using b actin as a second endogenous control.

### 4.9. Statistical Analysis

All data are expressed as arithmetic mean ± SD. Before the statistical analysis, we used the Shapiro–Wilk test (RStudio, Boston, MA, USA; URL http://www.rstudio.com/) and confirmed that all data had a normal distribution. Statistical analysis was then conducted using one-way ANOVA followed by Tukey’s post-test. The results were considered significant when the *p*-value was < 0.05.

## Figures and Tables

**Table 1:** Definition of experimental groups.

Group	Oral Administration	IP Injection
Control	PBS	PBS
T cell	PBS	T cell transfer
AgTc	Parasite extract	PBS
T cell+AgTc	Parasite extract	T cell transfer

**Table 2:** Definition of experimental groups.

		Adoptive T Lymphocyte Transfer	
Group	Parasite Extract by Gavage	Activated Splenic from wild type (WT)mice	Unstimulated Hepatic from GFP mice
A	Yes	No	No
B	Yes	Yes	No
C	Yes	Yes	Yes

## Supplementary Materials

The following are available online at https://www.mdpi.com/2076-0817/9/9/717/s1, Figure S1: Hepatic T lymphocytes isolation, Figure S2: Acetaminophen injection and liver damage.

## References

[1] Jenne C.N., Kubes P. (2013). Immune surveillance by the liver. Nat. Immunol..

[2] Krenkel O., Tacke F. (2017). Liver macrophages in tissue homeostasis and disease. Nat. Rev. Immunol..

[3] Poisson J., Lemoinne S., Boulanger C., Durand F., Moreau R., Valla D., Rautou P.E. (2017). Liver sinusoidal endothelial cells: Physiology and role in liver diseases. J. Hepatol..

[4] Yamamoto S., Sato Y., Shimizu T., Halder R.C., Oya H., Bannai M., Suzuki K., Ishikawa H., Hatakeyama K., Abo T. (1999). Consistent infiltration of thymus-derived T cells into the parenchymal space of the liver in normal mice. Hepatology.

[5] Lumsden A.B., Henderson J.M., Kutner M.H. (1988). Endotoxin levels measured by a chromogenic assay in portal, hepatic and peripheral venous blood in patients with cirrhosis. Hepatology.

[6] Renno T., Attinger A., Rimoldi D., Hahne M., Tschopp J., MacDonald H.R. (1998). Expression of B220 on activated T cell blasts precedes apoptosis. Eur. J. Immunol..

[7] Le Gall S.M., Legrand J., Benbijja M., Safya H., Benihoud K., Kanellopoulos J.M., Bobé P. (2012). Loss of P2X7 receptor plasma membrane expression and function in pathogenic B220+ double-negative T lymphocytes of autoimmune MRL/lpr mice. PLoS ONE.

[8] Mohamood A.S., Bargatze D., Xiao Z., Jie C., Yagita H., Ruben D., Watson J., Chakravarti S., Schneck J.P., Hamad A.R. (2008). Fas-mediated apoptosis regulates the composition of peripheral alphabeta T cell repertoire by constitutively purging out double negative T cells. PLoS ONE.

[9] Masztalerz A., Everse L.A., Otter W.D. (2004). Presence of cytotoxic B220+CD3+CD4-CD8- cells correlates with the therapeutic efficacy of lymphoma treatment with IL-2 and/or IL-12. J. Immunother..

[10] Bleesing J.J., Janik J.E., Fleisher T.A. (2003). Common expression of an unusual CD45 isoform on T cells from patients with large granular lymphocyte leukaemia and autoimmune lymphoproliferative syndrome. Br. J. Haematol..

[11] Hamad M. (2008). The case for extrathymic development of vaginal T lymphocytes. J. Reprod. Immunol..

[12] WHO Chagas-Disease-(American-Trypanosomiasis). https://www.who.int/news-room/fact-sheets/detail/chagas-disease-.

[13] Nogueira de Melo A.C., Meirelles M.N.N., Porrozzi R., Costa J.D., Branquinha M.H., Vermelho A.B.B. (2004). Reduced activity of matrix metalloproteinase-9 in *Trypanosoma cruzi*-infected mouse embryo hepatocyte cell. Hepatol. Res..

[14] Shimizu T., Bannai M., Kawamura H., Yamamoto S., Oya H., Maruyama S., Minagawa M., Kawamura T., Watanabe H., Hatakeyama K. (2000). Organ specificity of c-kit+ lymphoid precursors in the liver, thymus, and bone marrow. Eur. J. Haematol..

[15] Corbeaux T., Hess I., Swann J.B., Kanzler B., Haas-Assenbaum A., Boehm T. (2010). Thymopoiesis in mice depends on a Foxn1-positive thymic epithelial cell lineage. Proc. Natl. Acad. Sci. USA.

[16] Sardinha L.R., Elias R.M., Mosca T., Bastos K.R., Marinho C.R.R., D’Império Lima M.R., Alvarez J.M. (2006). Contribution of NK, NK T, gamma delta T, and alpha beta T cells to the gamma interferon response required for liver protection against *Trypanosoma cruzi*. Infect. Immun..

[17] Yamada H., Matsuzaki G., Chen Q., Iwamoto Y., Nomoto K. (2001). Reevaluation of the origin of CD44(high) “memory phenotype” CD8 T cells: Comparison between memory CD8 T cells and thymus-independent CD8 T cells. Eur. J. Immunol..

[18] Simon S., Labarriere N. (2017). PD-1 expression on tumor-specific T cells: Friend or foe for immunotherapy?. Oncoimmunology.

[19] Sauzay C., Voutetakis K., Chatziioannou A., Chevet E., Avril T. (2019). CD90/Thy-1, a Cancer-Associated Cell Surface Signaling Molecule. Front. Cell Dev. Biol..

[20] Kumar A., Bhanja A., Bhattacharyya J., Jaganathan B.G. (2016). Multiple roles of CD90 in cancer. Tumour Biol..

[21] Meuser-Batista M., Vacani-Martins N., Cascabulho C.M., Beghini D.G., Henriques-Pons A. (2020). In the presence of *Trypanosoma cruzi* antigens, activated peripheral T lymphocytes retained in the liver induce a proinflammatory phenotypic and functional shift in intrahepatic T lymphocyte. J Leukoc. Biol..

[22] Meuser-Batista M., Corrêa J.R., Carvalho V.F., de Carvalho Britto C.F., Moreira O.C., Batista M.M., Soares M.J., Filho F.A.E., Silva P.M., Lannes-Vieira J. (2011). Mast cell function and death in *Trypanosoma cruzi* infection. Am. J. Pathol..

[23] Birge R.B., Boeltz S., Kumar S., Carlson J., Wanderley J., Calianese D., Barcinski M., Brekken R.A., Huang X., Hutchins J.T. (2016). Phosphatidylserine is a global immunosuppressive signal in efferocytosis, infectious disease, and cancer. Cell Death Differ..

[24] Baaten B.J., Tinoco R., Chen A.T., Bradley L.M. (2012). Regulation of Antigen-Experienced T Cells: Lessons from the Quintessential Memory Marker CD44. Front. Immunol..

[25] Walling B.L., Kim M. (2018). LFA-1 in T Cell Migration and Differentiation. Front. Immunol..

[26] May H.D., Desai S.P., Kinjyo I., Harris J., Adams S.F. (2019). CD49d (high) T cells in the ovarian cancer microenvironment are a potential target for the optimization of immune checkpoint therapy in ovarian cancer. J. Clin. Oncol..

[27] Sardinha L.R., Mosca T., Elias R.M., do Nascimento R.S., Gonçalves L.A., Bucci D.Z., Marinho C.R., Penha-Gonçalves C., Lima M.R., Alvarez J.M. (2010). The liver plays a major role in clearance and destruction of blood trypomastigotes in *Trypanosoma cruzi* chronically infected mice. PLoS Negl. Trop. Dis..

[28] Mateus J., Guerrero P., Lasso P., Cuervo C., González J.M., Puerta C.J., Cuéllar A. (2019). An Animal Model of Acute and Chronic Chagas Disease With the Reticulotropic Y Strain of. Front. Immunol..

