# Supplementary material for: After Experimental Trypanosoma cruzi Infection, Dying Hepatic CD3+TCRαβ+B220+ T Lymphocytes Are Rescued from Death by Peripheral T Cells and Become Activated
Source: Pathogens. 2020 Aug 31;9(9):717. doi: 10.3390/pathogens9090717 (PMC7559066; doi:10.3390/pathogens9090717)
Supplement: Supplementary file 1 [file pathogens-09-00717-s001.pdf]

Supplementary Materials

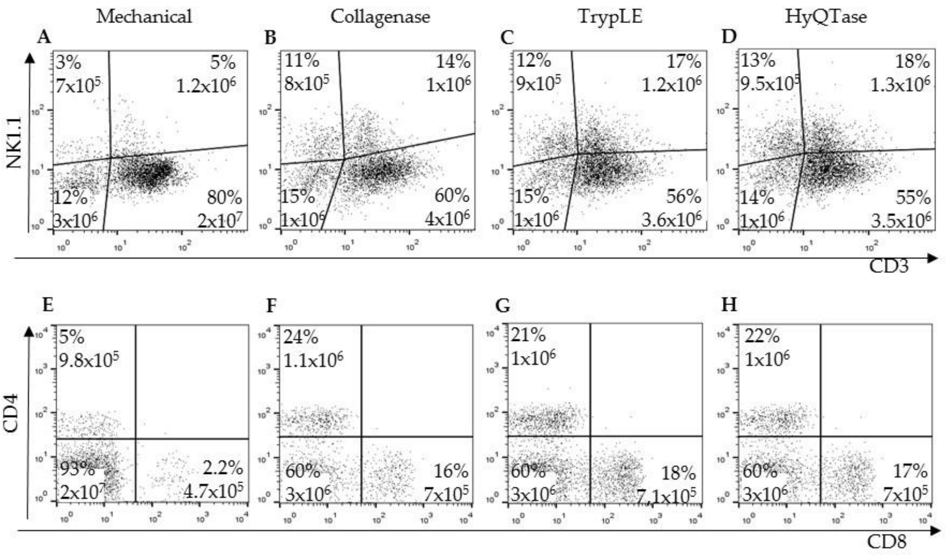

Figure S1. Hepatic T lymphocytes isolation. Thirteen-week old C57BL/10 mice were perfused through the hepatic portal vein, and the cells were obtained by mechanical dissociation (**A, E, I–K**); collagenase (type II, 120U/mL) (**B** and **F**); TrypLE (**C** and **G**), or HyQTase (**D** and **H**) for flow cytometry analysis. The analysis of NK (NK1.1^{+}CD3^{-}), NKT (NK1.1^{+}CD3^{+}), and CD3^{+} T lymphocytes (**A–D**) was done in the lymphocyte gate, while the analysis of CD4 and CD8 T lymphocytes was done in the gate of CD3^{+} T cells using the different methods (**E–H**). The relative (percentage) number of cells per sample is indicated in each quadrant. Five mice were pooled per group in at least four independent experiments.

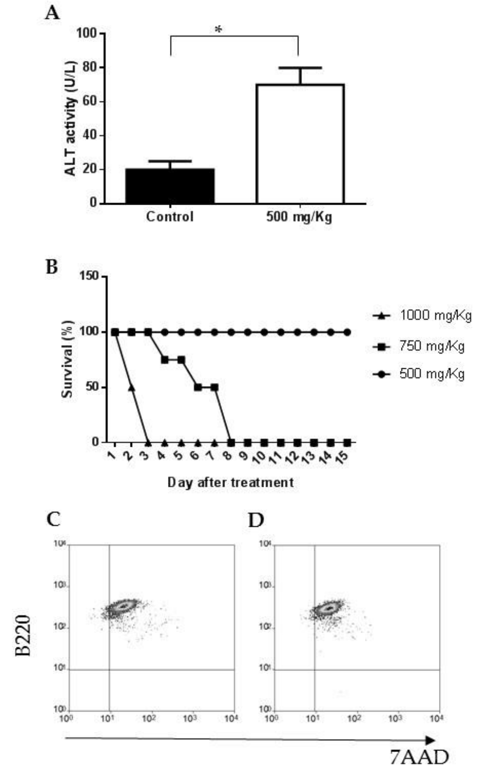

Figure S2. Acetaminophen injection and liver damage. Thirteen-week old C57BL/10 mice were IP injected with 500, 750, or 1000 mg/Kg of Acetaminophen. Blood ALT activity (**A**), mice survival (**B**), and the labeling of B220 and 7AAD in $CD3^{+}CD117^{+}$ T lymphocyte gate was evaluated in control (**C**) or mice treated with 500mg/Kg of Acetaminophen (**D**). Five mice were pooled per group in five independent experiments.
